# Comparison contrast-enhanced CT with contrast-enhanced US in diagnosing combined hepatocellular-cholangiocarcinoma: a propensity score-matched study

**DOI:** 10.1186/s13244-023-01576-6

**Published:** 2024-02-14

**Authors:** Jie Yang, Yun Zhang, Wu-yong-ga Bao, Yi-di Chen, Hanyu Jiang, Jia-yan Huang, Ke-yu Zeng, Bin Song, Zi-xing Huang, Qiang Lu

**Affiliations:** 1 Department of Medical Ultrasound, West China Hospital, Sichuan University, Chengdu, 610041 Sichuan China; 2 Department of Radiology, West China Hospital, Sichuan University, Chengdu, 610041 Sichuan China; 3 Department of Radiology, Sanya People’s Hospital, Hainan, China; 4 Department of Radiology, West China Tianfu hospital of Sichuan University, Sichuan, China

**Keywords:** Liver neoplasms, Ultrasonography, Tomography (X-ray Computed), Diagnosis (Differential)

## Abstract

**Objectives:**

To develop and compare noninvasive models for differentiating between combined hepatocellular-cholangiocarcinoma (cHCC-CCA) and HCC based on serum tumor markers, contrast-enhanced ultrasound (CEUS), and computed tomography (CECT).

**Methods:**

From January 2010 to December 2021, patients with pathologically confirmed cHCC-CCA or HCC who underwent both preoperative CEUS and CECT were retrospectively enrolled. Propensity scores were calculated to match cHCC-CCA and HCC patients with a near-neighbor ratio of 1:2. Two predicted models, a CEUS-predominant (CEUS features plus tumor markers) and a CECT-predominant model (CECT features plus tumor markers), were constructed using logistic regression analyses. Model performance was evaluated by the area under the curve (AUC), sensitivity, specificity, and accuracy.

**Results:**

A total of 135 patients (mean age, 51.3 years ± 10.9; 122 men) with 135 tumors (45 cHCC-CCA and 90 HCC) were included. By logistic regression analysis, unclear boundary in the intratumoral nonenhanced area, partial washout on CEUS, CA 19-9 > 100 U/mL, lack of cirrhosis, incomplete tumor capsule, and nonrim arterial phase hyperenhancement (APHE) volume < 50% on CECT were independent factors for a diagnosis of cHCC-CCA. The CECT-predominant model showed almost perfect sensitivity for cHCC-CCA, unlike the CEUS-predominant model (93.3% vs. 55.6%, *p* < 0.001). The CEUS-predominant model showed higher diagnostic specificity than the CECT-predominant model (80.0% vs. 63.3%; *p* = 0.020), especially in the ≤ 5 cm subgroup (92.0% vs. 70.0%; *p* = 0.013).

**Conclusions:**

The CECT-predominant model provides higher diagnostic sensitivity than the CEUS-predominant model for CHCC-CCA. Combining CECT features with serum CA 19-9 > 100 U/mL shows excellent sensitivity.

**Critical relevance statement:**

Combining lack of cirrhosis, incomplete tumor capsule, and nonrim arterial phase hyperenhancement (APHE) volume < 50% on CECT with serum CA 19-9 > 100 U/mL shows excellent sensitivity in differentiating cHCC-CCA from HCC.

**Key points:**

1. Accurate differentiation between cHCC-CCA and HCC is essential for treatment decisions.

2. The CECT-predominant model provides higher accuracy than the CEUS-predominant model for CHCC-CCA.

3. Combining CECT features and CA 19-9 levels shows a sensitivity of 93.3% in diagnosing cHCC-CCA.

**Graphical Abstract:**

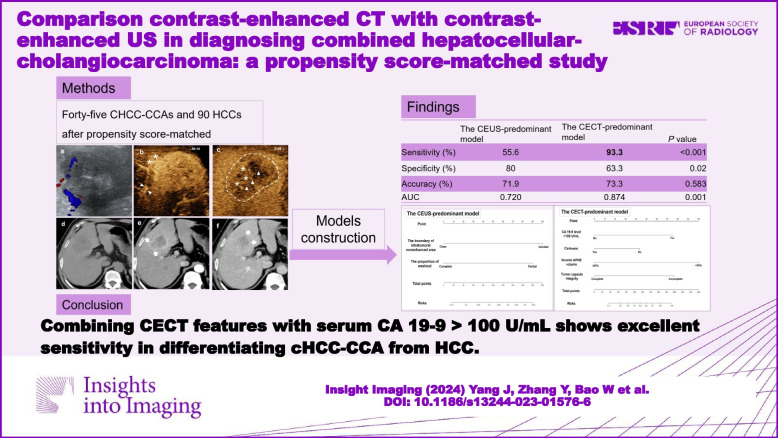

**Supplementary Information:**

The online version contains supplementary material available at 10.1186/s13244-023-01576-6.

## Introduction

Combined hepatocellular-cholangiocarcinoma (cHCC-CCA) accounts for 0.4–4.2% of primary liver cancer cases and demonstrates hepatocytic and biliary differentiation in the same tumor [1]. In routine practice, a marked overlap in clinical conditions has been observed between cHCC-CCA and hepatocellular carcinoma (HCC) [2, 3].

However, the treatment strategies for cHCC-CCA and HCC differ. For example, liver transplantation has been accepted as an effective curative-intent treatment option for HCC, but it is not recommended for cHCC-CCA because of frequent recurrence (54% at 5 years) and suboptimal long-term survival (41% at 5 years) [4–6]. Lymph node dissection is recommended for resectable cHCC-CCA, while it is not routinely performed for HCC [1, 7]. Local treatment and systematic therapy are established treatment options for unresectable HCCs [8, 9], but whether they have therapeutic benefits for unresectable cHCC-CCAs is controversial [6, 10]. Thus, the accurate differential diagnosis between cHCC-CCA and HCC is critical for appropriate therapeutic decision-making.

Contrast-enhanced ultrasound (CEUS) and contrast-enhanced computed tomography (CECT) are two of the main imaging modalities for diagnosing liver tumors [11, 12], and each of these modalities has different imaging principles and advantages. CEUS is performed with pure blood contrast and can continuously evaluate macro- and microvascular perfusion within tumors [13]. CECT uses a small molecule contrast agent that can assess the entire liver during a standard examination and has demonstrated clear advantages over CEUS in detecting tumors and extrahepatic lymph metastases [14]. Previous studies have explored the potential of combining laboratory results with CEUS or CECT for differentiating cHCC-CCA from HCC but have yielded suboptimal diagnostic performances (sensitivity for cHCC-CCA: 32.5 to 74.4%) [2, 15–18]. No studies have compared the diagnostic efficacy of CEUS and CECT in differentiating cHCC-CCA from HCC. It is also uncertain which imaging examination is best for diagnosing probable HCC/cHCC-CCA in high-risk patients.

Here, we aimed to develop diagnostic models integrating clinical and readily accessible CEUS and CECT features to differentiate between cHCC-CCA and HCC in a propensity score-matched study and to compare the two models.

## Patients and methods

### Patients

**Fig. 1:**
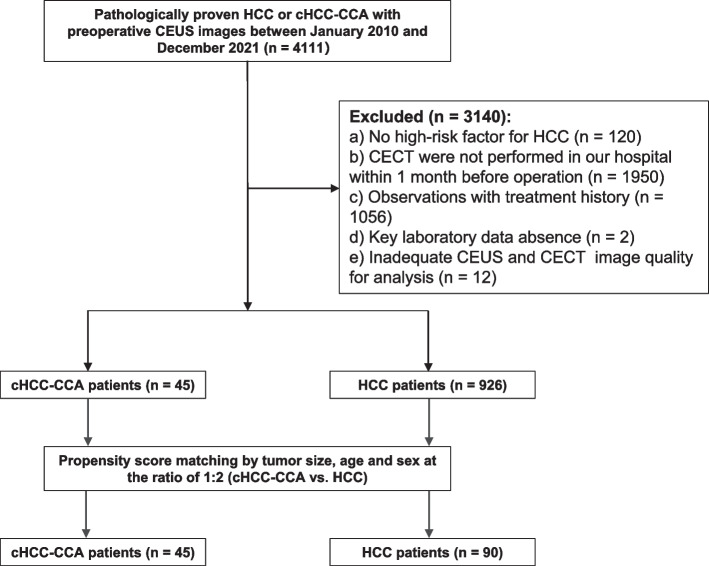
Flowchart of included patients. cHCC-CCA, combined hepatocellular-cholangiocarcinoma; HCC, hepatocellular carcinoma; CEUS, contrast-enhanced ultrasound; CECT, contrast-enhanced computed tomography

From January 2010 to December 2021, patients who underwent curative-intent liver resection for surgically proven HCC or cHCC-CCA were consecutively enrolled. The inclusion criteria were as follows: (a) pathologically proven HCC or cHCC-CCA, (b) both CECT and CEUS examinations within 1 month before surgery, and (c) chronic hepatitis B/C virus infection or cirrhosis. Patients were excluded if (a) they had received any prior antitumoral treatment, (b) key laboratory data were not available, or (c) CECT and/or CEUS images were degraded or missing. The inclusion and exclusion flowchart is shown in Fig. 1.

### Imaging acquisition

The imaging acquisition recommendation and the detailed parameters are presented in eMethods 1 in Supplement 1.

### Imaging analysis

All image analyses were conducted on a per-lesion basis by two ultrasonographers (K-Y.Z. and J-Y.H., with 8 and 10 years of experience in CEUS images, respectively) and two radiologists (Y.Z. and Y-D.C., with 8 and 11 years of experience in liver imaging in CECT images, respectively). All disagreements between the reviewers regarding the imaging features were resolved by consensus. For patients with multiple lesions, the largest targeted lesion was selected for feature-related analyses.

### CEUS

**Fig. 2:**
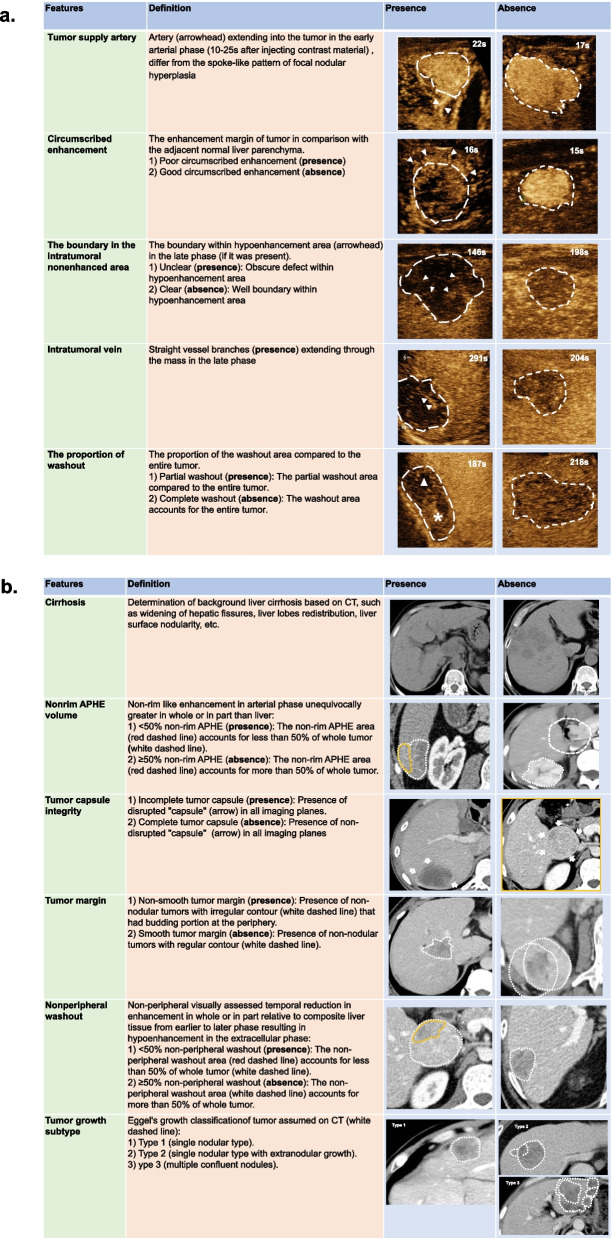
The definition of the partial imaging features of the lesions on CEUS (**a**) and CECT (**b**)

The CEUS imaging features and LI-RADS categories according to ACR CEUS LI-RADS version 2017 [19], along with tumor size, number, cirrhosis, enhanced level in the arterial phase/portal venous phase/late phase (AP/PVP/LP), etc., were evaluated. The following non-LI-RADS imaging features that have been associated with cHCC-CCA or HCC were also evaluated: (a) tumor supply artery: present vs. absent [20, 21]; (b) circumscribed enhancement: poor vs. good [20, 21]; (c) the boundary in the intratumoral nonenhanced area: clear vs. unclear [21]; (d) the proportion of washout: partial vs. complete; and (e) intratumoral vein: present vs. absent [21–23]. The detailed definitions of the imaging features are presented in eMethods 2 in Supplement 1 and typical cases are shown in Fig. 2a.

### CECT

The CECT imaging features and LI-RADS categories according to ACR CECT LI-RADS version 2018 [24], along with tumor size and number, were evaluated. The following non-LI-RADS imaging features that have been associated with cHCC-CCA or HCC were also evaluated: (a) cirrhosis: present vs. absent [25]; (b) semiquantitative characteristics for quantifying hypervascular components, including the nonrim arterial phase hyperenhancement (APHE) volume ratio (< 50% vs. ≥ 50%) and nonperipheral washout volume ratio (< 50% vs. ≥ 50%); (c) tumor capsule integrity: complete vs. incomplete [7]; (d) tumor margin: smooth vs. nonsmooth [26]; and (e) tumor growth subtype (Eggel’s growth classification as assumed on CT): type 1 (single nodular type), type 2 (single nodular type with extranodular growth), and type 3 (multiple confluent nodules) [27]. The detailed definitions of the imaging features are presented in eMethods 2 in Supplement 1 and typical cases are shown in Fig. 2b.

### Histopathology analysis

The pathological characteristics of the lesions were retrospectively recorded according to the pathological reporting system in our hospital. These included the maximum size of the main lesion (the largest lesion in the case of multiple lesions), the Edmondson-Steiner grade of the HCC, and the HCC/intrahepatic cholangiocarcinoma (ICC)-predominant components of cHCC-CCA [28].

### Statistical analysis

Propensity score-matching was performed to minimize the effect of potential selection bias and confounding factors between patients with HCC and cHCC-CCA.

The predictive models, based separately on tumor markers and CEUS and CECT features, were constructed using logistic regression analyses, and their performance was compared with that of pathology. The variables with *p* < 0.05 by the *χ*^2^ test or Fisher’s exact test were entered into the univariate logistic analysis, and the multicollinearity between the univariate variables was assessed using Spearman’s correlation analysis and by computing the variance inflation factor (VIF). If the absolute value of the correlation coefficient (ACC) was ≥ 0.6 or the VIF was > 10 between two variables, the variable with the higher odds ratio (OR) were selected for multivariate logistic analysis. Therefore, all independent variables that were associated with cHCC-CCA in univariate analyses were input into a multivariate logistic regression model using the backward stepwise method while adjusting for the same covariates as above. The diagnostic models were illustrated as nomograms based on their correlation coefficients in the multivariate logistic analysis.

Model discrimination was assessed by computing the area under the receiver operating characteristic curve (AUC) value and compared using the DeLong test. Model calibration was evaluated by the Hosmer–Lemeshow (H-L) test and calibration curves. The McNemar test was used to compare pairwise sensitivities, specificities, and accuracies of the two diagnostic models. The subgroup comparison of the diagnostic efficacy between CEUS and CECT was also evaluated for smaller lesions, with a diameter of 5 cm.

All statistical analyses were performed with the R software (R Foundation for Statistical Computing, version 3.2.5, http://www.r-project.org/) and MedCalc (version 10.4). A two-tailed adjusted *p <* 0.05 was statistically significant.

## Results

### Patients

A total of 971 patients were initially identified. After propensity score matching, 135 patients (mean age, 51.3 ± 10.9 years, 122 males [90.4%]) with 135 nodules (45 cHCC-CCAs and 90 HCCs) were included for further analysis.

Serum CA 19-9 > 100 U/mL was more frequently found in patients with cHCC-CCA than in those with HCC (11.1% vs. 2.2%, *p =* 0.029), while serum AFP > 400 μg/L was more frequently observed in patients with HCC than in those with cHCC-CCA (36.7% vs. 20.0%, *p =* 0.050). The key clinical features of the patients are summarized in Table 1.

**Table 1:** Basic clinical and pathological characteristics of patients with cHCC-CCA and HCC

Characteristics	cHCC-CCA (*n* = 45)	HCC (*n* = 90)	*p* value^#^
**Patients**^a^			
Age (years)	52 ± 9.2	51 ± 11.7	0.630
Sex			0.681
Male	40 (88.9)	82 (91.1)	
Female	5 (11.1)	8 (8.9)	
Hepatitis status			1.000
HBV (+)	44 (97.8)	87 (96.7)	
HCV (+)	1 (2.2)	1 (1.1)	
Others	0 (0)	2 (2.2)	
AFP level (μg/L)			0.050
0–400	36 (80.0)	57 (63.3)	
> 400	9 (20.0)	33 (36.7)	
CA 19-9 level (U/mL)			0.029
0–100	40 (88.9)	88 (97.8)	
> 100	5 (11.1)	2 (2.2)	
**Pathological characteristics of the main mass**^b^			
Size (cm)	5.6 ± 3.8	5.5 ± 3.5	0.851
Size (cm)			0.807
≤ 5	26 (57.8)	50 (55.6)	
> 5	19 (42.2)	40 (44.6)	
Edmondson-Steiner grade			< 0.001
1	1 (2.2)	2 (2.2)	
2	3 (6.7)	50 (55.6)	
3	11 (24.4)	14 (15.6)	
Both 2–3^c^	7 (15.6)	23 (25.6)	
Not available	23 (51.1)	1 (1.1)	

*cHCC-CCA* combined hepatocellular-cholangiocarcinoma, *HCC* hepatocellular carcinoma, *HBV* hepatitis B virus, *HCV* hepatitis C virus, *AFP* alpha-fetoprotein, *CA 19-9* carbohydrate antigen 19-9

^#^Clinical variables were subjected to further logistic regression analysis when *p* values were < 0.05 in the *χ*^2^ test or Fisher’s exact test, as appropriate

^a^Unless stated otherwise, data in parentheses are counts (percentages)

^b^For the multiple nodules in the liver, only the largest nodule was analyzed in this study

^c^The components of HCC were included in both grades 2 and 3 based on pathological results

### Imaging features and interrater agreement

Based on the CEUS LI-RADS classification, 46.6% and 37.8% of cHCC-CCA patients were classified as LR-4/5 and LR-M, respectively; for HCC, 65.6% and 20% were classified as LR-4/5 and LR-M, respectively. Based on the CECT LI-RADS classification, 44.5% and 51.1% of cHCC-CCA patients were classified as LR-4/5 and LR-M, respectively, compared with 76.7% for LR-4/5 and 12.2% for LR-M among the HCC patients.

On CEUS, the following features were more frequent in patients with cHCC-CCA than in those with HCC: hypoenhancement in the PVP images (88.9% vs. 64.4%), unclear boundary in the intratumoral nonenhanced area (71.1% vs. 37.8%), and partial washout (71.1% vs. 40.0%). The baseline CEUS imaging features of all lesions are presented in Table 2.

**Table 2:** The CEUS features of included lesions

Imaging features	cHCC-CCA^a^ (*n* = 45)	HCC (*n* = 90)	*p* value^#^
***B-model ultrasound***			
Size (cm)	5.6 ± 3.5	5.8 ± 3.8	0.799
Number of tumors (single)	18 (40.0)	26 (28.9)	0.243
Cirrhosis	23 (51.1)	43 (47.8)	
Nodule echo (hypo-)	40 (88.9)	68 (75.6)	0.109
Boundary (well)	13 (28.9)	35 (38.9)	0.170
Shape (regular)	16 (35.6)	45 (50.0)	0.143
***CEUS***			
Enhancement level in the AP			1.000
Hyperenhancement	44 (97.8)	89 (98.9)	
Isoenhancement	1 (2.2)	1 (1.1)	
Hypoenhancement	0 (0.0)	0 (0.0)	
Enhancement level in the PVP			0.002
Hyperenhancement	0 (0.0)	0 (0.0)	
Isoenhancement	5 (11.1)	32 (35.6)	
Hypoenhancement	40 (88.9)	58 (64.4)	
Enhancement level in the LP			0.424
Hyperenhancement	0 (0.0)	0 (0.0)	
Isoenhancement	1 (2.2)	6 (6.7)	
Hypoenhancement	44 (97.8)	84 (93.3)	
*LI-RADS major features*			
Rim APHE	1 (2.3)	1 (1.1)	1.000
Early washout	19 (42.4)	23 (25.6)	0.075
Marked washout within two minutes	0 (0.0)	1 (1.1)	1.000
Mild and late washout	44 (97.8)	84 (93.3)	1.000
Tumor in vein	7 (15.6)	13 (14.4)	1.000
LI-RADS category			0.113
LR-4	1 (2.2)	6 (6.7)	
LR-5	20 (44.4)	53 (58.9)	
LR-M	17 (37.8)	18 (20)	
LR-TIV	7 (15.6)	13 (14.4)	
*LI-RADS ancillary features*			
Nodule-in-nodule architecture	0 (0.0)	0 (0.0)	NA
Mosaic architecture	9 (20.0)	14 (15.6)	0.628
*Other features*			
Tumor supply artery	24 (53.3)	44 (48.9)	0.716
Circumscribed enhancement (well)	20 (44.4)	45 (50.0)	0.587
Unclear boundary in the intratumoral nonenhanced area	32 (71.1)	34 (37.8)	< 0.001
Intratumoral vein in LP	12 (26.7)	17 (18.9)	0.206
The proportion of washout (partial)	32 (71.1)	36 (40.0)	< 0.001
Necrosis or severe ischemia	4 (8.9)	17 (18.9)	0.207

*cHCC-CCA* combined hepatocellular-cholangiocarcinoma, *HCC* hepatocellular carcinoma, *CEUS* contrast-enhanced ultrasound, *AP* arterial phase, *PVP* portal venous phase, *LP* late phase, *NA* not available, *APHE* arterial phase hyperenhancement, *LI-RADS* liver imaging reporting and data system, *LR* liver imaging reporting and data system category

^#^Categorical variables were compared by the *χ*^2^ test or Fisher’s exact test

^a^Unless otherwise indicated, data are number of patients, with percentage in parentheses

On CECT, the following features were more commonly observed in cHCC-CCA: nonrim APHE volume < 50% (57.8% vs. 12.2%), rim APHE (37.8% vs. 5.6%), nonperipheral washout volume < 50% (48.9% vs. 20.0%), peripheral washout (48.9% vs. 8.9%), LR-M category (51.1% vs. 12.2%), and incomplete tumor capsule (60.0% vs. 40.0%). The following features were more frequently detected for HCC: cirrhosis (68.9% vs. 44.4%) and single nodular type (tumor growth subtype 1) (68.9% vs. 51.1%). The baseline CECT imaging features of all lesions are presented in Table 3.

**Table 3:** The CECT features of included lesions

Imaging features	cHCC-CCA^a^ (*n* = 45)	HCC (*n* = 90)	*p* value^#^
***CT scan***			
Size (cm)	5.5 ± 3.6	5.3 ± 3.4	0.719
Number of tumors (single)	33 (73.3)	82 (80.0)	0.511
Cirrhosis	20 (44.4)	62 (68.9)	0.009
***CECT***			
*LI-RADS major features*			
Nonrim APHE volume (< 50%)	26 (57.8)	11 (12.2)	< 0.001
Rim APHE	17 (37.8)	5 (5.6)	< 0.001
Nonperipheral washout volume (< 50%)	22 (48.9)	18 (20.0)	< 0.001
Peripheral washout	22 (48.9)	8 (8.9)	< 0.001
Enhancing capsule	28 (62.2)	52 (57.8)	0.621
Tumor in vein	1 (2.2)	8 (9.0)	0.169
LI-RADS category			< 0.001
LR-3	1 (2.2)	2 (2.2)	
LR-4	8 (17.8)	8 (8.9)	
LR-5	12 (26.7)	61 (67.8)	
LR-M	23 (51.1)	11(12.2)	
LR-TIV	1 (2.2)	8 (8.9)	
*LI-RADS ancillary features*			
Corona enhancement	11 (24.4)	21 (23.3)	0.889
Nonenhancing capsule	4 (8.9)	17 (18.9)	0.132
Nodule-in-nodule architecture	9 (20.0)	23 (25.6)	0.526
Mosaic architecture	11 (24.4)	30 (33.3)	0.326
Blood products in mass	2 (4.4)	5 (5.6)	1.000
Fat in mass, more than adjacent liver	0 (0.0)	1 (1.1)	1.000
Delayed central enhancement	2 (4.4)	7 (7.8)	0.464
Internal artery	14 (31.1)	35 (38.9)	0.449
Necrosis or severe ischemia	18 (40.0)	48 (53.3)	0.201
Infiltrative appearance	20 (44.4)	38 (42.2)	0.855
*Other features*			
Tumor capsule integrity (incomplete)	27 (60.0)	36 (40.0)	0.044
Tumor margin (smooth)			0.855
Tumor growth subtype			< 0.001
Type 1: single nodular type	23 (51.1)	62 (68.9)	
Type 2: single nodule with extranodular growth	12 (26.7)	26 (28.9)	
Type 3: multiple confluent nodules	10 (22.2)	2 (2.2)	
Lesion with LR-M features^b^	24 (53.3)	11 (12.2)	< 0.001

*cHCC-CCA* combined hepatocellular-cholangiocarcinoma, *HCC* hepatocellular carcinoma, *CECT* contrast-enhanced computed tomography, *APHE* arterial phase hyperenhancement, *LI-RADS* liver imaging reporting and data system, *LR* liver imaging reporting and data system category

^#^Categorical variables were compared by the *χ*^2^ test or Fisher’s exact test

^a^Unless otherwise indicated, data are number of patients, with percentage in parentheses

^b^Lesion with LR-M features means that lesions have LR-M category features with or without tumor thrombus in a vessel on CECT

Cohen’s kappa values ranged from 0.312 to 0.765 for CEUS and from 0.380 to 0.717 for CECT. The interrater agreement of imaging features on CEUS and CECT are summarized in eTable 3 in Supplement 1.

### The efficiency of the imaging models for cHCC-CCA

#### The CEUS-predominant model

**Fig. 3:**
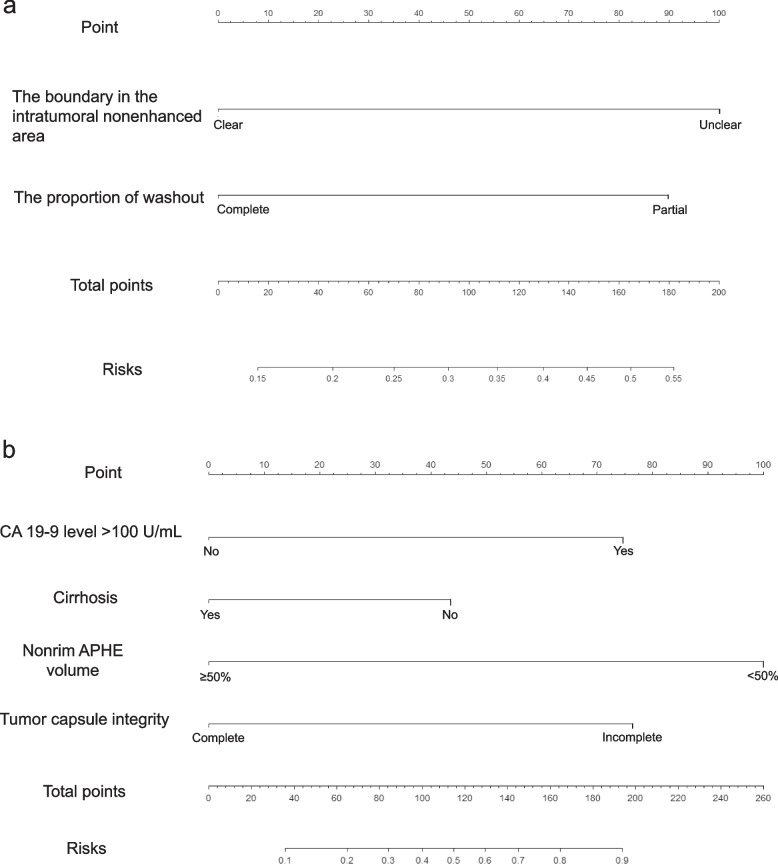
Nomograms of the CEUS-predominant (**a**) and CECT-predominant models (**b**)

The CEUS-predominant model was developed by combining CEUS features and tumor markers (AFP > 400 μg/L and CA 19-9 > 100 U/mL). The univariate variable selection is presented in eMethods 3 in Supplement 1. By multivariate regression analysis, unclear boundary in the intratumoral nonenhanced area (OR = 2.765; 95% confidence interval [CI]: 1.209, 6.541; *p* = 0.018) and partial washout (OR = 2.607; 95% CI: 1.152, 6.079; *p* = 0.023) were independent factors for a diagnosis of cHCC-CCA (shown in Table 4). The AUC value of the prediction model was 0.720 (95% CI: 0.632, 0.808). The sensitivity, specificity, and accuracy were 55.6%, 80.0%, and 71.9%, respectively. Regression coefficient-based nomograms were constructed based on the CEUS-predominant model (Fig. 3a). The calibration curve of the nomogram for the probability of cHCC-CCA demonstrated good agreement between prediction and observation (eFigure 1a). The H-L test yielded a nonsignificant statistic (*p* = 1.000).

**Table 4:** Univariate and multivariate logistic regression analyses for diagnosing cHCC-CCA with the CEUS-predominant and CECT-predominant models

Variables	Univariable analysis			Multivariable analysis		
	*β*	OR (95% CI)	*p* value	*β*	OR (95% CI)	*p* value
***The CEUS-predominant model***						
CA19-9 level > 100 (U/mL)	1.705	5.500 (1.023, 29.567)	0.047	..	..	..
Unclear boundary in the intratumoral nonenhanced area	1.4	4.054 (1.872, 8.780)	< 0.001	1.017	2.765 (1.209, 6.541)	0.018
Partial washout	1.306	3.692 (1.709, 7.977)	0.001	0.958	2.607 (1.152, 6.079)	0.023
Hypoenhancement in the PVP	− 1.485	0.227 (0.081, 0.631)	0.045	..	..	..
***The CECT-predominant model***						
CA19-9 level > 100 (U/mL)	1.705	5.500 (1.023, 29.567)	0.047	2.149	8.573 (1.217, 82.845)	0.038
Cirrhosis	− 1.018	0.361 (0.173, 0.756)	0.007	− 1.179	0.308 (0.113, 0.795)	0.017
Rim APHE^a^	2.334	10.324 (3.489, 30.538)	< 0.001	..	..	..
Peripheral washout	2.104	8.200 (3.222, 20.871)	< 0.001	..	..	..
Nonrim APHE volume < 50%	2.285	9.828 (4.139, 23.335)	< 0.001	2.414	11.180 (3.475, 41.419)	< 0.001
Nonperipheral washout volume < 50%	1.342	3.826 (1.754, 8.347)	0.001	..	..	..
Incomplete tumor capsule	1.325	3.763 (1.771, 7.997)	0.001	1.944	7.348 (2.394, 25.929)	< 0.001
Tumor growth subtype 2 or 3^b^	− 0.750	0.472 (0.225, 0.984)	0.045	..	..	..
Lesion with LR-M features^c^	2.105	8.208 (3.471, 19.410)	< 0.001	..	..	..

*cHCC-CCA* combined hepatocellular-cholangiocarcinoma, *CEUS* contrast-enhanced ultrasound, *CECT* contrast-enhanced computed tomography, *CA 19-9* carbohydrate antigen 19-9, *OR* odds ratio, *PVP* portal venous phase, *APHE* arterial phase hyperenhancement, *CI* confidence interval, *LR* liver imaging reporting and data system category

^a^Collinearity exists among these variables (the same words on the right upper), variables with higher odds ratio were used by multivariate regression analysis

^b^Tumor growth subtype: type 2 (single nodule with extranodular growth); type 3 (multiple confluent nodules)

^c^Lesion with LR-M features means that lesions have LR-M category feature accompanying with/without tumor thrombus in vascular on CECT

#### The CECT-predominant model

The CECT-predominant model was developed by combining CECT features and tumor markers. The univariate variable selection is presented in eMethods 3 in Supplement 1. On multivariate regression analysis, CA 19-9 > 100 U/mL (OR = 8.573; 95% CI: 1.217, 82.845; *p* = 0.038), cirrhosis (OR = 0.308; 95% CI: 0.113, 0.795; *p* = 0.017), incomplete tumor capsule (OR = 7.348; 95% CI: 2.394, 25.929; *p* < 0.001), and nonrim APHE volume < 50% (OR = 11.180; 95% CI, 3.475, 41.419; *p* < 0.001) were found to be independent factors for diagnosing cHCC-CCA (shown in Table 4). The AUC value of the prediction model was 0.874 (95% CI: 0.816, 0.931), with a sensitivity, specificity, and accuracy of 93.3%, 63.3%, and 73.3%, respectively. A regression coefficient-based nomogram was constructed based on the CECT-predominant model (Fig. 3b). The calibration curve of the nomogram for the probability of cHCC-CCA demonstrated good agreement between prediction and observation (eFigure 1b). The H-L test yielded a nonsignificant statistic (*p* > 0.05).

### Comparison between the imaging models

**Fig. 4:**
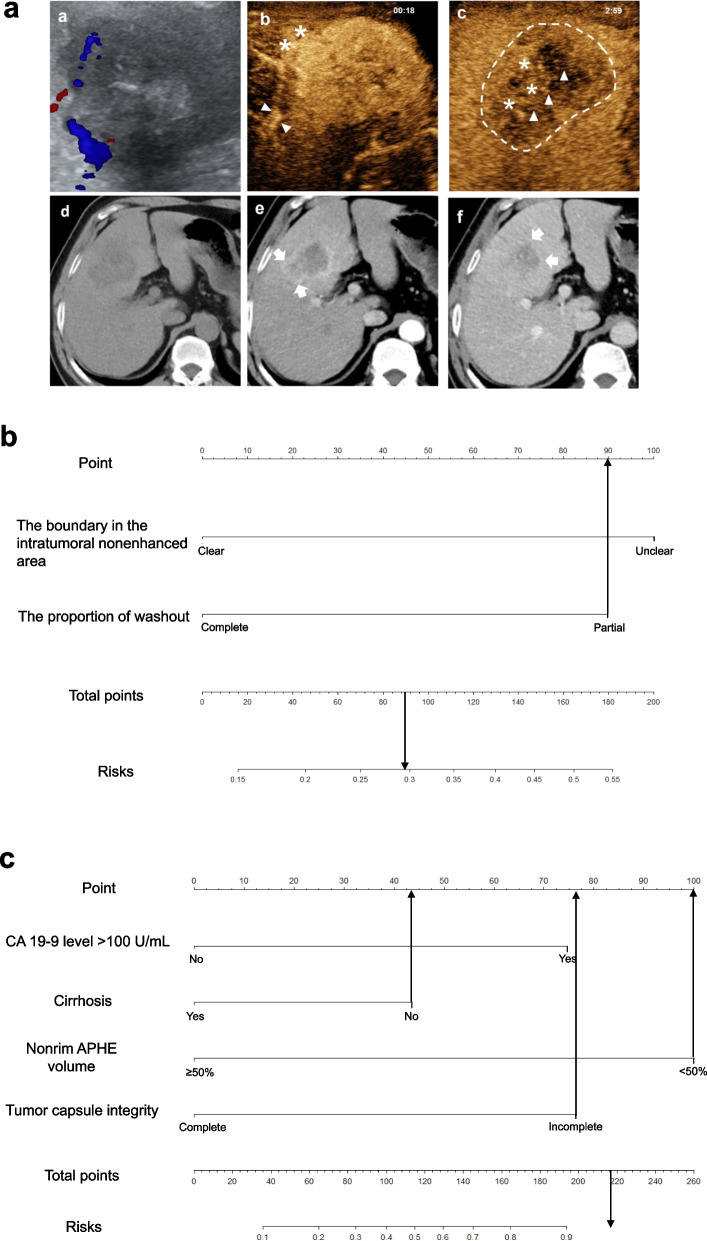
CEUS and CECT images of a 56-year-old man with chronic hepatitis B and CA 19-9 < 100 U/mL. A 7.3-cm mass was detected in segment IV of the liver (**A**). A hypoechoic mass with poor boundary on conventional ultrasound (**A**, a); on CEUS, the mass showed hyperenhancement, a nonsmooth tumor margin (stars), and tumor supply artery (arrowhead) at 18 s (**A**, b); in the late phase (179 s), the hyperenhanced area in the arterial phase of mass exhibited partial washout with partial isoenhancement (stars) and partial hypoenhancement area (arrowhead, **A**, c). Based on these features, the likelihood of this mass being diagnosed as cHCC-CCA was smaller than 30% according to the CEUS-predominant model (**B**). There was no obvious cirrhotic liver background, and the mass showed low density on abdominal CT image (**A**, d), rim enhancement and < 50% nonrim enhancement (mainly the right posterior part of the lesion, arrow) in the arterial phase (**A**, e), “washout” absence, nonsmooth tumor margin, and a thin incomplete enhanced capsule (arrow) seen in the portal venous phase (**A**, f). Based on these features, the likelihood of this mass being diagnosed as cHCC-CCA was higher than 90.0% according to the CECT-predominant model (**C**). The mass was pathologically proven to be combined hepatocellular-cholangiocarcinoma

**Fig. 5:**
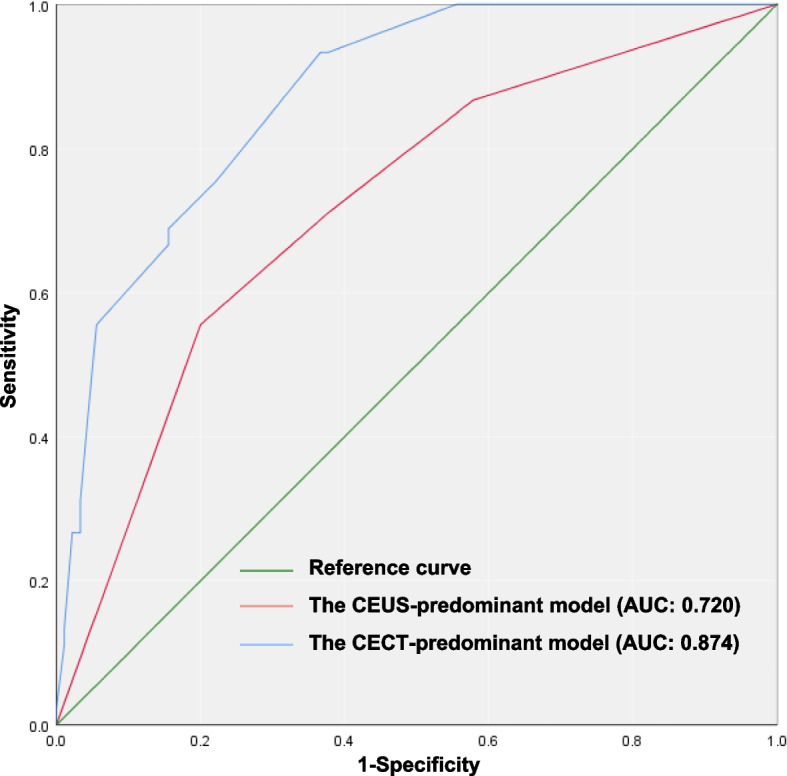
The diagnostic performance of the CEUS-predominant and CECT-predominant models was assessed through ROC curve and AUC analyses

The diagnostic performance was compared between the CEUS-predominant model and the CECT-predominant model (shown in Table 5 and Fig. 4). The CECT-predominant model had a higher diagnostic sensitivity (93.3%) than the CEUS-predominant model (55.6%; *p* < 0.001) but a lower diagnostic specificity (CECT vs. CEUS: 63.3% vs. 80.0%; *p* = 0.020). The two models had comparable diagnostic accuracy (CECT vs. CEUS: 73.3% vs. 71.9%; *p* = 0.583). In addition, we compared the AUC values between the models and found that the AUC value of the CECT-predominant model (AUC_CECT_ = 0.874, 95% CI: 0.816, 0.931) was higher than that of the CEUS-predominant model (AUC_CEUS_ = 0.720, 95% CI: 0.632, 0.808; *p* = 0.001, Fig. 5).

**Table 5:** Comparison of the diagnostic performance between the CEUS-predominant and CECT-predominant models

	The CEUS-predominant model	The CECT-predominant model	*p value*^*#*^
**Total**			
Sensitivity (%)	55.6	93.3	< 0.001
Specificity (%)	80.0	63.3	0.020
Accuracy (%)	71.9	73.3	0.583
AUC (95% CI)^a^	0.720 (0.632, 0.808)	0.874 (0.816, 0.931)	0.001
≤ **5 (cm)**			
Sensitivity (%)	50.0	88.5	0.006
Specificity (%)	92.0	70.0	0.013
Accuracy (%)	77.6	76.3	1.000
AUC (95% CI)	0.710 (0.595, 0.808)	0.792 (0.684, 0.877)	0.226
**> 5 (cm)**			
Sensitivity (%)	63.2	100	0.016
Specificity (%)	65.0	55.0	0.503
Accuracy (%)	64.4	69.5	0.557
AUC (95% CI)	0.641 (0.505, 0.762)	0.775 (0.648, 0.873)	0.093

*CEUS* contrast-enhanced ultrasound, *CECT* contrast-enhanced computed tomography, *AUC* area under the curve, *CI* confidence interval

^a^The discrimination of AUC value was considered fair (AUC < 0.6), moderate (AUC, 0.6-0.75), and substantial (AUC > 0.75), respectively

^#^*p* value was obtained from the comparison between the CEUS-predominant and CECT-predominant models by the McNemar test. *p* < 0.05 was considered to indicate a statistical difference

### Subgroup analysis for the CEUS-predominant and CECT-predominant models

For the smaller nodules (≤ 5 cm, based on the pathology results) group, the CECT-predominant model had higher diagnostic sensitivity for cHCC-CCA than the CEUS-predominant model (88.5% vs. 50.0%; *p* = 0.006), while the CEUS-predominant model presented better diagnostic specificity than the CECT-predominant model (92.0% vs. 70.0%; *p* = 0.013). The two models showed comparable diagnostic performance in differentiating cHCC-CCA from HCC (AUC_CECT_ = 0.792 vs. AUC_CEUS_ = 0.710; *p* = 0.226, shown in eFigure 2a). In the > 5 cm subgroup, the CECT-predominant model had perfect diagnostic sensitivity for cHCC-CCA (100% vs. 63.2% of the CEUS-predominant model; *p* = 0.016). Its ROC curve is shown in eFigure 2b. The detailed diagnostic performance is shown in Table 5.

## Discussion

Combining tumor biomarkers and imaging features is critical in diagnosing cHCC-CCA due to its overlapping features with HCC. This propensity score-matched study found that approximately 44.4% of cHCC-CCAs on CEUS and 26.7% of cHCC-CCAs on CECT were evaluated as LR-5, which can easily mimic HCC. Therefore, we constructed and compared two imaging-predominant diagnostic models based on clinical data and nodule features on CEUS and CECT imaging to identify cHCC-CCA. The results indicated that the CECT-predominant model exhibited nearly perfect diagnostic sensitivity (93.3%), which was significantly higher than that of the CEUS-predominant model (55.6%; *p* < 0.001). On the other hand, the CEUS-predominant model demonstrated commendable diagnostic specificity, particularly for lesions smaller than 5 cm (92.0% vs. 70.0%; *p* = 0.013).

Cirrhosis detected by CECT is highly suggestive of HCC. In this study, we found that few at-risk patients with cHCC-CCA had a cirrhotic liver background due to the different origins of HCC and cHCC-CCA, which is similar to the findings of the latest studies [24, 29–31]. Additionally, this study revealed that cHCC-CCA patients exhibited higher rates of nonrim APHE with a volume < 50%. This finding is congruent with a previous study that reported a larger HCC component (*p* = 0.014) and a smaller ICC component (*p* = 0.001) in the hypervascular group of cHCC-CCA patients during pathological analysis [32]. In addition, capsular enhancement is usually considered a major imaging feature of HCC [24, 33]. In the present study, we observed a higher frequency of incomplete tumor capsules in cHCC-CCA than in HCCs. Similar to the observation of “unclear boundaries” on CEUS, the presence of an incomplete capsule is likely associated with infiltrative tumor growth of the ICC portion in cHCC-CCA [15, 34]. Interestingly, ten out of 12 cHCC-CCAs, classified as LR-5 based on CECT LI-RADS classification, were indeed reclassified as cHCC-CCA based on the CECT-predominant model in this study, which may help improve the diagnostic specificity of LR-5 for HCC in future clinical practice.

On CEUS images, the presence of unclear boundaries in the intratumoral nonenhanced areas was an independent risk factor for cHCC-CCA. This might be elucidated by the fibrotic pathological findings (relying on the ICC component), similar to previous findings [21, 35]. The presence of washout on CEUS with SonoVue reflects the intratumoral vascular supply. Therefore, ICCs often present earlier and with more complete washout compared to HCCs [36]. Consequently, partial washout could frequently be observed in cHCC-CCA lesions that contain both HCC and ICC components. Notably, tumor differentiation is correlated with the presence of washout, as demonstrated by the findings of Iavarone et al. [37]. This study also observed that grade 2–3 HCCs exhibited higher rates of partial washout than lower grade HCCs (grade 1 or 2) (19/37 vs. 17/52; *p* = 0.079). This finding might provide valuable prognostic information for future studies in this field.

Several studies have evaluated the performance of imaging characteristics in differentiating cHCC-CCA and HCC in recent years [15, 18, 31, 38, 39]. The model developed in this study, which combined CECT features and CA 19-9 levels to distinguish cHCC-CCA, was put into a visual form as a nomogram and demonstrated a remarkable sensitivity of 93.3%, showing better performance than previous ones (ranging from 40 to 73.8% [15, 18, 38]. We hope that our findings can offer valuable guidance in two aspects. First, the remarkably high sensitivity for cHCC-CCA could effectively diagnose lesions that do not support a definite diagnosis of HCC, which might improve the diagnostic specificity of HCC in routine clinical practice. In addition, the CECT-predominant model exhibited a low specificity of 63.3%, which could lead to misdiagnosis or underdiagnosis of a higher number of HCC cases, which would limit the therapeutic options for HCC (e.g., liver transplantation). Given this, adding CEUS to CECT could improve the overall diagnostic accuracy, especially for lesions less than 5 cm.

Some limitations of this study should be mentioned. First, there was an unavoidable selection bias due to the single-center retrospective nature of the study, although we used PSM to lessen this bias. Second, no validation data were available to test and refine our models due to the limited size of the cHCC-CCA population. Third, we did not include ICC patients in this differential diagnostic study due to the limited number of ICC patients with HCC risk factors. Finally, the results of this study were based on a case-control design rather than a cohort design, which might not reflect real-world clinical epidemiological conditions. Therefore, large-scale multicenter studies are warranted to validate our findings.

## Conclusions

The CECT-predominant model provides higher diagnostic sensitivity compared to the CEUS-predominant model for cHCC-CCA. Combining the CECT features with serum CA 19-9 > 100 U/mL showed excellent diagnostic sensitivity in differentiating cHCC-CCA from HCC, while the CEUS features could enhance diagnostic specificity, especially in the ≤ 5 cm subgroup.

### Supplementary Information

**Additional file 1: Supplement 1: eMethods 1.** Imaging acquisition protocols **eMethods 2.** Variable definition **eMethods 3.** The univariable selection of the CEUS-predominant and the CECT-predominant model **eTable 1.** Vascular phases of the liver lesions on CEUS **eTable 2.** Multi-phase contrast-enhanced CT scan **eTable 3.** The kappa analysis of imaging features assessment on CEUS and CECT between reviewers **eTable 4.** The multicollinearity analysis between variables of the CEUS-predominant model **eTable 5.** The multicollinearity analysis between variables of the CECT-predominant model **eFigure 1.** The calibration analysis of the CEUS-predominant model and the CECT-predominant models **eFigure 2.** The ROC curve of the two models in the subgroup analysis.

## Data Availability

The datasets used or analyzed during the current study are available from the corresponding author upon reasonable request.

## Abbreviations

AFP: Alpha-fetoprotein
APHE: Arterial phase hyperenhancement
CA 19-9: Carbohydrate antigen 19-9
CECT: Contrast-enhanced computed tomography
CEUS: Contrast-enhanced ultrasound
cHCC-CCA: Combined hepatocellular-cholangiocarcinoma
CI: Confidence interval
HBV: Hepatitis B virus
HCC: Hepatocellular carcinoma
HCV: Hepatitis C virus
LI-RADS: Liver Imaging Reporting and Data System
LR: Liver Imaging Reporting and Data System category
OR: Odds ratio
PSM: Propensity score matching
PVP: Portal venous phase
TIV: Tumor in vein
