# Supplementary material for: Comparison contrast-enhanced CT with contrast-enhanced US in diagnosing combined hepatocellular-cholangiocarcinoma: a propensity score-matched study
Source: Insights Imaging. 2024 Feb 14;15:44. doi: 10.1186/s13244-023-01576-6 (PMC10866845; doi:10.1186/s13244-023-01576-6)
Supplement: Supplementary file 1 — Additional file 1: Supplement 1: eMethods 1. Imaging acquisition protocols eMethods 2. Variable definition eMethods 3. The univariable selection of the CEUS-predominant and the CECT-predominant model eTable 1. Vascular phases of the liver lesions on CEUS eTable 2. Multi-phase contrast-enhanced CT scan eTable 3. The kappa analysis of imaging features assessment on CEUS and CECT between reviewers eTable 4. The multicollinearity analysis between variables of the CEUS-predominant model eTable 5. The multicollinearity analysis between variables of the CECT-predominant model eFigure 1. The calibration analysis of the CEUS-predominant model and the CECT-predominant models eFigure 2. The ROC curve of the two models in the subgroup analysis. [file 13244_2023_1576_MOESM1_ESM.pdf]

Comparison contrast-enhanced CT with contrast-enhanced US in diagnosing combined hepatocellular-cholangiocarcinoma: A propensity score-matched study

ELECTRONIC SUPPLEMENTARY MATERIAL

Content:

eMethods 1. Imaging acquisition protocols

eMethods 2. Variable definition

eMethods 3. The univariable selection of the CEUS-predominant and the CECT-predominant model

eTable 1. Vascular phases of the liver lesions on CEUS

eTable 2. Multi-phase contrast-enhanced CT scan

eTable 3. The kappa analysis of imaging features assessment on CEUS and CECT between reviewers

eTable 4. The multicollinearity analysis between variables of the CEUS-predominant model

eTable 5. The multicollinearity analysis between variables of the CECT-predominant model

eFigure 1. The calibration analysis of the CEUS-predominant model and the CECT-predominant models

eFigure 2. The ROC curve of the two models in the subgroup analysis.

eMethods 1: Imaging acquisition protocols

CEUS

The contrast-enhanced ultrasound (CEUS) examination was acquired after detecting liver lesions on US images with a convex or line array transducer. Contrast-specific pulse inversion harmonic imaging using a mechanical index of less than 0.1 was used for all CEUS examinations by following the World Federation for Ultrasound in Medicine and Biology and the European Federation of Societies for Ultrasound in Medicine and Biology (WFUMB-EFSUMB) technical recommendations [1]. A bolus injection of 1.2-2.4 mL ultrasound agent SonoVue (Bracco, Milan, Italy) was performed through a 20-gauge line placed in the antecubital vein, followed by a flush of 5 mL 0.9% sodium chloride solution. The imaging timer was started simultaneously with the injection of SonoVue.

All patients underwent CEUS examinations by experienced ultrasound physicians using a Resona 7 system (Mindray, Shenzhen, China) equipped with an SC6-1U abdominal convex probe, an iU22 system (Philips Bothell, Washington, USA) equipped with a C5-1 convex array transducer or an EPIQ 7 system (Philips Bothell, Washington, USA) equipped with a C5-1 convex array transducer.

Image recording was performed continuously from the first bubble arrival through peak AP enhancement as a minimum requirement and followed the recommendations of the 2017 version of the Liver Imaging Reporting and Data System (LI-RADS) (http://www.acr.org/quality-safety/resources/LI-RADS). Optionally, the cine loop could be continued beyond the AP enhancement peak until 60s after injection. After 60 s, the recording of static images at 60 s and with every intermittent (every 30–60 s) acquisition thereafter is sufficient to document and evaluate the presence, timing, and degree of washout [2]. Detailed scan time-range suggestions are summarized in eTable 1.

eTable 1. Vascular phases of the liver lesions on CEUS.

Phase	Start (s)	End (s)
AP	10-20	30-45
PVP	30-45	120
LP	>120	Bubble disappearance (approx. 4-6 min)

AP: arterial phase; PVP: portal venous phase; LP: late phase.

CECT

The contrast-enhanced CT images were acquired on various CT systems. All the patients underwent multiphase contrast-enhanced CT scans by using Omnipaque (350; GE Healthcare) contrast medium. Omnipaque was intravenously injected at a rate of 3 mL/s. The trigger threshold of the aorta reached 100 HU. Arterial phase (AP) and portal venous phase (PVP) were performed at 35 s, and 70 s, respectively, after intravenous injection of contrast. If the lesion showed continuous enhancement on PVP images, the delayed phase (120 s after intravenous injection of contrast) was added. Detailed scanning parameters are summarized in eTable 2.

eTable 2. Multiphase contrast-enhanced CT scan.

**Scan models**	**kV (voltage) pre/AP/PVP**	**Rotation time (s)**	**Single collimation width**	**Section thickness (mm) pre/AP/PVP**
Siemens Definition	120/120/120	0.5	1.2	7/5/5
Siemens SOMATOM Definition AS+	120/120/120	0.5	0.6	5/5/2
Siemens SOMATOM Definition Flash	100/120/120	0.5	0.6	5/2/2

AP: arterial phase; PVP: portal venous phase.

eMethods 2. Variable definition

The ultrasound features were defined as follows:

B-mode ultrasound features

(1) Number of tumors: 1) single: solitary tumor; 2) multiple (tumor number ≥2).
(2) Tumor size (cm): maximum diameter of the targeted nodule.
(3) Cirrhosis: if the whole liver presented with surface nodularity, overall coarse and heterogeneous echotexture, segment hypertrophy, splenomegaly, ascites and so on.
(4) Nodule echo: hypoechoic or hyperechoic contrast to adjacent liver parenchyma (hypoecho/hyperecho).
(5) Shape of the nodule (regular/irregular).
(6) Boundary of the nodule (well-defined/poor).

CEUS features

(7) Enhancement level of the nodule in the arterial (AP)/portal venous (PVP)/late phase (LP) (hyper/iso/hypo).
(8) Necrosis or thrombus area: defined as irregular area with no contrast enhancement in all three phases.
(9) Major LI-RADS features: [2]
    1) Rim APHE: enhancement mainly concentrated at the periphery of the liver nodule (indicates ring-like hyperenhancement in the arterial phase).
    2) Early washout: timing of the first observation of washout appears less than 60 s after contrast bolus injection.
    3) Mild and late washout: the presence of washout appears after more than 60 s and the degree of washout is mild.
    4) Marked washout: "punched-out" within 120 s after contrast agent injection.
    5) Tumor in vein: defined as tissue-like hyperenhancement followed by washout regardless of the onset and degree of washout in the vein.
(10) Ancillary features:
    1) Mosaic architecture in the AP: refers to the presence within a mass of randomly distributed internal nodules differing in echogenicity on B-mode imaging and contrast enhancement intensity on CEUS.
    2) Nodule-in-nodule architecture: increased enhancement of one or more inner nodules within a larger enhancing nodule.
(11) Non-LI-RADS imaging features:
    1) Tumor supply artery: Artery extending into the tumor in the early arterial phase (10-25 s after injecting contrast material), differs from the spoke-like pattern of

focal nodular hyperplasia.

2) Circumscribed enhancement: The enhancement margin of the tumor in comparison with the adjacent normal liver parenchyma (poor/good).
3) The boundary in the intratumoral nonenhanced area: The boundary within the hypoenhancement area in the late phase (unclear/clear).
4) Intratumoral vein: straight vessel branches extending through the mass in the late phase.
5) The proportion of washout: the proportion of the washout area compared to the entire tumor (partial/complete).

Of note, if unsure about the presence of any of the abovementioned features, we regarded the feature as absent.

The CT features were characterized as follows:

CT scan features

(1) Tumor size: largest outer-edge-to-outer-edge dimension of an observation.

(2) Number of tumors: 1) single: solitary tumor; 2) multiple (tumor number ≥2).

CECT features

(3) LI-RADS major features [3]

1) Rim APHE: spatially defined subtype of APHE in which arterial phase enhancement is most pronounced in the observation periphery.
2) Peripheral washout: spatially defined subtype of “washout” in which apparent washout is most pronounced in the observation periphery.
3) Nonrim APHE: nonrim like enhancement in the AP unequivocally greater in whole or in part than the liver:
   a. Nonrim APHE <50%: the nonrim APHE area accounts for less than 50% of whole tumor.
   b. Nonrim APHE ≥50%: the nonrim APHE area accounts for more than 50% of whole tumor.
4) Nonperipheral washout: Nonperipheral visually assessed temporal reduction in enhancement in whole or in part relative to composite liver tissue from earlier to later phase resulting in hypoenhancement in the extracellular phase:
   a. Nonperipheral washout <50%: the nonperipheral washout area accounts for less than 50% of whole tumor.
   b. Nonperipheral washout ≥50%: the nonperipheral washout area accounts for more than 50% of whole tumor.
5) Enhancing capsule: smooth, uniform, sharp border around most or all of a liver observation, unequivocally thicker or more conspicuous than fibrotic

tissue around background nodules, and visible as enhancing rim in portal venous phase.

(4) LI-RADS ancillary features [3]

1) Corona enhancement: peri-observational enhancement in the late arterial phase or early portal venous phase, attributable to venous drainage from the tumor.

2) Nonenhancing capsule: capsule appearance not visible as an enhancing rim.

3) Nodule-in-nodule architecture: presence of a smaller inner nodule within and having different imaging features than a larger outer nodule.

4) Mosaic architecture: presence of randomly distributed internal nodules or compartments, usually with different imaging features.

5) Blood products in mass: blood products in a mass, in absence of biopsy, trauma or intervention.

6) Fat in mass, more than adjacent liver: more fat in a mass than in liver.

7) Delayed central enhancement: central area of progressive postarterial phase enhancement.

8) Internal artery: presence of discrete arterial enhancement within the tumor

9) Necrosis or severe ischemia: presence of unequivocal intralesional necrosis or severe ischemia.

10) Infiltrative appearance: observation with a noncircumscribed margin (indistinct transition) thought to represent malignancy with permeative growth pattern.

(5) Other features

1) Cirrhosis: determination of background liver cirrhosis based on CT images, such as widening of hepatic fissures, liver lobe redistribution, and liver surface nodularity [4].

2) Tumor capsule integrity [5; 6]: a. incomplete: presence of disrupted "capsule" in all imaging planes; b. complete: presence of nondisrupted "capsule" in all imaging planes.

3) Tumor margin [7]: a. nonsmooth tumor margin: presence of nonnodular tumors with irregular contours that had budding portions at the periphery; b. smooth tumor margin: presence of non-nodular tumors with regular contours.

4) Tumor growth subtype: Eggel's growth classification of tumor assumed on CT [8]: a. type 1 (single nodular type); b. type 2 (single nodular type with extranodular growth); c. type 3 (multiple confluent nodules).

5) Tumor in vein: presence of unequivocal enhancing soft tissue in vein [3].

eMethods 3. The univariable selection of the CEUS-predominant and CECT-predominant models

The CEUS-predominant model

For the CEUS-predominant model, CA19-9 level >100 U/mL, unclear boundary in the intratumoral nonenhanced area, partial washout, and hypoenhancement in the PVP were identified as significant predictors for a cHCC-CCA diagnosis in univariate analysis. Multicollinearity analysis between univariate variables was subsequently conducted (shown in eTable 4). The above variables were thus all included in multivariate regression analysis.

The CECT-predominant model

For the CECT-predominant model, multiple features, including CA19-9 level >100 U/mL, cirrhosis, rim APHE, peripheral washout, nonrim APHE volume <50%, nonperipheral washout volume <50%, incomplete tumor capsule, tumor growth subtype 2 or 3, and lesion with LR-M features, were identified as significant predictors for a cHCC-CCA diagnosis in univariate analysis. The results of the subsequent multicollinearity analysis among univariate variables are listed in eTable 5. The variables (rim APHE and nonrim APHE volume <50%) with higher OR values were included in the multivariate regression analysis. The ACCs among the other variables were 0.010-0.579, and the VIF < 10. Then, CA 19-9 level >100 U/mL, cirrhosis, peripheral washout, nonrim APHE volume <50%, incomplete tumor capsule, tumor growth subtype 2 or 3, and lesion with LR-M features were included in the multivariate regression analysis.

eTable 3. The kappa analysis of imaging features assessment on CEUS and CECT between reviewers.

Imaging features	Kappa value*	Agreement#
**Ultrasound**		
B-model ultrasound		
Cirrhosis	0.511 (0.366, 0.656)	Moderate
Nodule echo (hypo-/hyper)	0.615 (0.455, 0.776)	Substantial
Boundary (well-defined/poor)	0.616 (0.477, 0.754)	Substantial
Shape (regular/irregular)	0.409 (0.256, 0.561)	Moderate
CEUS		
Enhancement level in the AP (hyper-/iso-/hypo-)	0.492 (0.114, 1.000)	Moderate
Enhancement level in the PVP (hyper-/iso-/hypo-)	0.686 (0.559, 0.812)	Substantial
Enhancement level in the LP (hyper-/iso-/hypo-)	0.687 (0.429, 0.944)	Substantial
LI-RADS major features		
Rim APHE	0.431 (0.029, 0.833)	Moderate
Early washout	0.634 (0.496, 0.772)	Substantial
Marked washout within two minutes	0.393 (0.147, 0.933)	Fair
Mild and late washout	0.687 (0.429, 0.944)	Substantial
Tumor in vein	0.496 (0.308, 0.685)	Moderate
LI-RADS category (LR-4/5/M/TIV)	0.607 (0.480, 0.733)	Substantial
LI-RADS ancillary features		
Nodule-in-nodule architecture†	--	--
Mosaic architecture	0.312 (0.199, 0.426)	Fair
Other features		
Tumor supply artery	0.524 (0.398, 0.651)	Moderate
Tumor margin (smooth/nonsmooth)	0.564 (0.433, 0.696)	Moderate
Unclear boundary in the intratumor nonenhanced area	0.491 (0.359, 0.623)	Moderate
Intratumoral vein	0.498 (0.311, 0.685)	Moderate
Partial washout	0.426 (0.294, 0.557)	Moderate
Necrosis or thrombus area	0.765 (0.610, 0.921)	Substantial
**Computed tomography**		
CT scan		
Cirrhosis	0.455 (0.301, 0.609)	Moderate
CECT		
LI-RADS major features		
Nonrim APHE volume ratio (<50%/≥50%)	0.649 (0.523, 0.776)	Substantial
Rim APHE	0.541 (0.367, 0.715)	Moderate

Nonperipheral washout volume ratio (<50%/≥50%)	0.511 (0.352, 0.670)	Moderate
Peripheral washout	0.539 (0.098, 0.779)	Moderate
Enhancing capsule	0.460 (0.328, 0.592)	Moderate
LI-RADS ancillary features		
Corona enhancement	0.382 (0.227, 0.537)	Fair
Nonenhancing capsule	0.380 (0.152, 0.609)	Fair
Nodule-in-nodule architecture	0.579 (0.448, 0.709)	Moderate
Mosaic architecture	0.395 (0.289, 0.501)	Fair
Blood products in mass	0.688 (0.433, 0.943)	Substantial
Fat in mass, more than adjacent liver	0.663 (0.044, 1.000)	Substantial
Delayed central enhancement	0.717 (0.481, 0.953)	Substantial
Internal artery	0.660 (0.538, 0.781)	Substantial
Necrosis or severe ischemia	0.652 (0.547, 0.758)	Substantial
Infiltrative appearance	0.579 (0.448, 0.709)	Moderate
Other features		
Tumor capsule integrity (complete/incomplete)	0.717 (0.481, 0.953)	Substantial
Tumor margin (smooth/non-smooth)	0.598 (0.466, 0.730)	Moderate
Tumor growth subtype (type 1/2/3) ‡	0.471 (0.352, 0.590)	Moderate
Tumor in vein	0.485 (0.263, 0.708)	Moderate
LI-RADS category (LR-3/4/5/M/TIV)	0.419 (0.286, 0.553)	Moderate

cHCC-CCA: combined hepatocellular-cholangiocarcinoma; HCC: hepatocellular carcinoma; CEUS: contrast-enhanced ultrasound; CECT: contrast-enhanced computed tomography; AP: arterial phase; PVP: portal venous phase; LP: late phase; APHE: arterial phase hyperenhancement; LI-RADS: Liver imaging reporting and data system; LR: Liver imaging reporting and data system classification.

* The consistency of the binary variables was evaluated by Cohen's kappa values, whereas the consistency of the multiple variables were evaluated by weighted kappa values.

# Inter-rater agreement was considered poor (κ < 0.2), fair (κ: 0.2-0.4), moderate (κ: 0.4-0.6), substantial (κ: 0.6-0.8) or almost perfect (κ: 0.8-1.0), respectively.

† No nodule-in-nodule architecture was evaluated in the 135 liver lesions according to the two reviewers.

‡ Tumor growth subtype: type 1 defined as single nodular type; type 2 defined as single nodular type with extra-nodular growth; and type 3 defined as multiple confluent nodules.

eTable 4. The multicollinearity analysis between variables of the CEUS-predominant model.

ACC*	CA 19-9 level > 100 U/mL	Hypoenhancement in the PVP	Unclear boundary in the intratumoral nonenhanced area	Partial washout
CA 19-9 level >100 U/mL	1.000	-0.069	0.039	0.098
Hypoenhancement in the PVP	..	1.000	-0.302	-0.187
Unclear boundary in the intratumoral non-enhanced area	..	..	1.000	0.289
Partial washout	..	..	..	1.000

ACC: the absolute value of correlation coefficient; CEUS: contrast-enhanced ultrasound; PVP: portal venous phase; CA 19-9: carbohydrate antigen 19-9

* If ACC ≥0.6, there were multicollinearity existence between variables.

eTable 5. The multicollinearity analysis between variables of the CECT-predominant model.

ACC*	CA 19-9 > 100 U/mL	Cirrhosis	Rim APHE	Peripheral washout	Nonrim APHE volume <50%	Nonperipheral washout volume <50%	Incomplete tumor capsule	Tumor growth subtype 2 or 3	Lesion with LR-M features
CA 19-9 level > 100 U/mL	1.000	0.051	0.078	0.045	0.081	0.068	0.028	-0.028	0.014
Cirrhosis	...	1.000	-0.179	-0.262	-0.152	-0.043	0.020	-0.083	-0.286
Rim APHE	..	..	1.000	0.714	0.583	0.504	0.077	-0.118	0.609
Peripheral washout	..	..	..	1.000	0.505	0.308	0.137	-0.024	0.448
Nonrim APHE volume <50%	..	..	..	..	1.000	0.656	-0.024	-0.010	0.470
Nonperipheral washout volume <50%	..	..	..	..	..	1.000	0.006	-0.006	0.579
Incomplete tumor capsule	..	..	..	..	..	..	1.000	-0.492	0.141
Tumor growth subtype 2 or 3[#]	..	..	..	..	..	..	..	1.000	-0.141
Lesion with LR-M features[†]	..	..	..	..	..	..	..	..	1.000

ACC: the absolute value of correlation coefficient; CECT: contrast-enhanced computed tomography; CA 19-9: carbohydrate antigen 19-9; APHE: arterial phase hyperenhancement; LR: Liver imaging reporting and data system classification

* If ACC ≥0.6 between variables, there were multicollinearity existence between variables.

[#] Tumor growth subtype 2 or 3: defined as single nodular type with extra-nodular growth or multiple confluent nodules

[†] Lesion with LR-M features means that lesions have LR-M category feature accompanying with/without tumor thrombus in vascular on CECT.

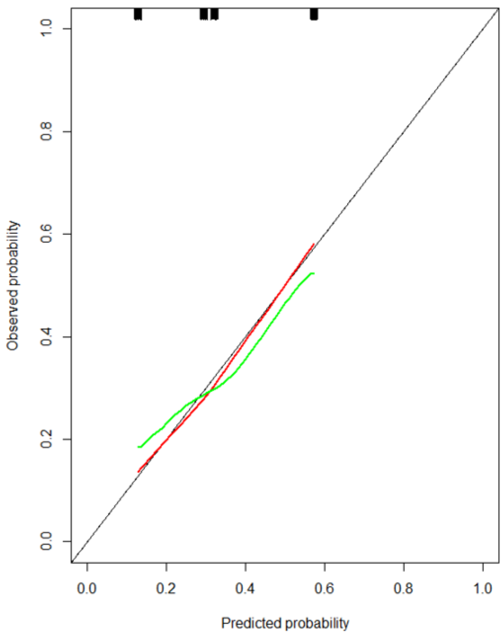

(a)

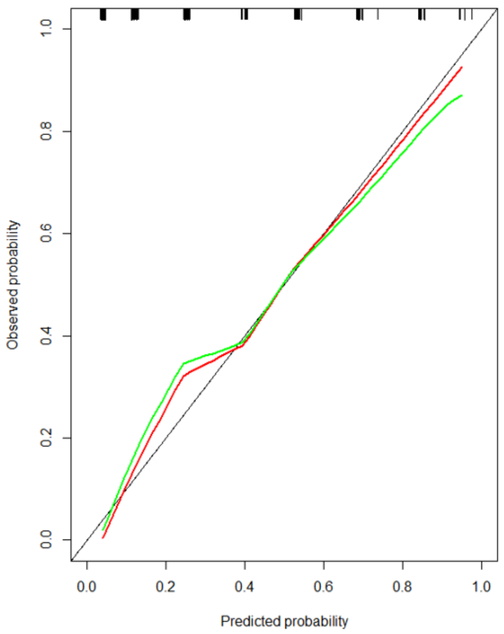

(b)

eFigure 1. The calibration analysis of the CEUS-predominant model and the CECT-predominant models**.** Calibration plots of the CEUS-predominant model (a), CECT-predominant model (b).

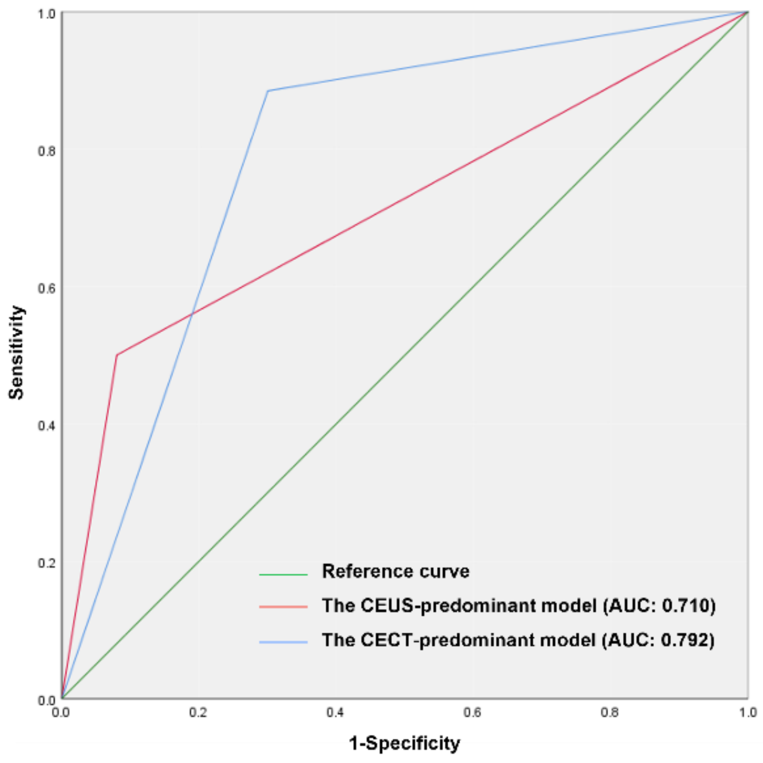

(a)

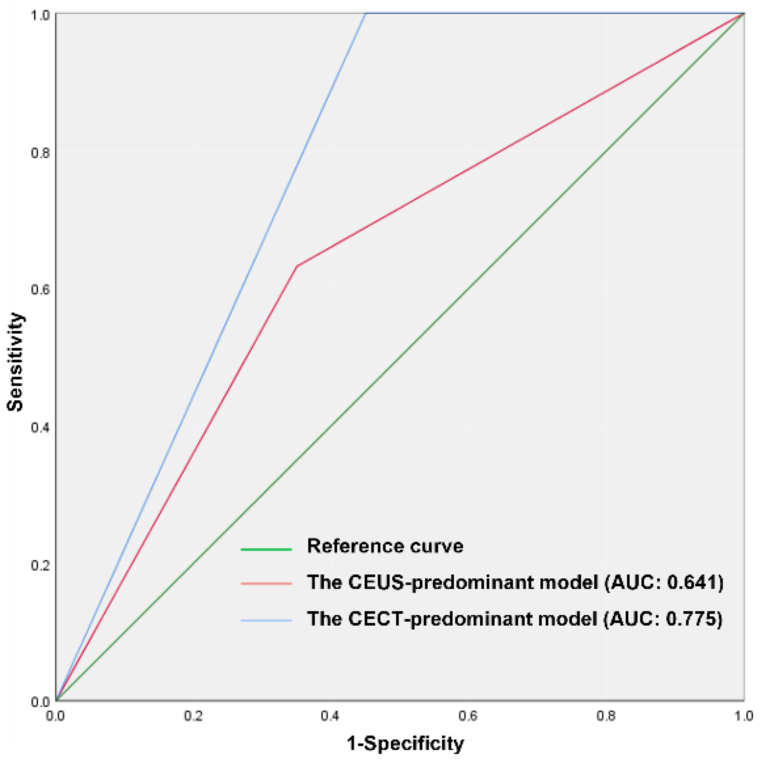

(b)

eFigure 2. The ROC curve of the two models in the subgroup analysis. The ROC curve of the two models in ≤5 cm (a) and >5 cm (b) subgroup.

References

1. Claudon M, Dietrich CF, Choi BI et al (2013) Guidelines and good clinical practice recommendations for Contrast Enhanced Ultrasound (CEUS) in the liver - update 2012: A WFUMB-EFSUMB initiative in cooperation with representatives of AFSUMB, AIUM, ASUM, FLAUS and ICUS. Ultrasound in Med & Biol 39:187-210. Doi:10.1016/j.ultrasmedbio.2012.09.002

2. Lyshchik A, Kono Y, Dietrich CF et al (2018) Contrast-enhanced ultrasound of the liver: technical and lexicon recommendations from the ACR CEUS LI-RADS working group. Abdominal Radiology (New York) 43:861-879. Doi:10.1007/s00261-017-1392-0

3. Chernyak V, Fowler KJ, Kamaya A et al (2018) Liver Imaging Reporting and Data System (LI-RADS) Version 2018: Imaging of Hepatocellular Carcinoma in At-Risk Patients. Radiology 289:816-830. Doi:10.1148/radiol.2018181494

4. Dodd GD, Baron RL, Oliver JH, Federle MP (1999) Spectrum of imaging findings of the liver in end-stage cirrhosis: part I, gross morphology and diffuse abnormalities. AJR American Journal of Roentgenology 173:1031-1036

5. Alhasan A, Cerny M, Olivié D et al (2019) LI-RADS for CT diagnosis of hepatocellular carcinoma: performance of major and ancillary features. Abdominal Radiology (New York) 44:517-528. Doi:10.1007/s00261-018-1762-2

6. Kim H, Park MS, Choi JY et al (2009) Can microvessel invasion of hepatocellular carcinoma be predicted by pre-operative MRI? Eur Radiol 19:1744-1751. Doi:10.1007/s00330-009-1331-8

7. Ariizumi S-i, Kitagawa K, Kotera Y et al (2011) A non-smooth tumor margin in the hepatobiliary phase of gadoxetic acid disodium (Gd-EOB-DTPA)-enhanced magnetic resonance imaging predicts microscopic portal vein invasion, intrahepatic metastasis, and early recurrence after hepatectomy in patients with hepatocellular carcinoma. Journal of Hepato-biliary-pancreatic Sciences 18:575-585. Doi:10.1007/s00534-010-0369-y

8. Hui AM, Takayama T, Sano K et al (2000) Predictive value of gross classification of hepatocellular carcinoma on recurrence and survival after hepatectomy. J Hepatol 33:975-979. Doi:10.1016/s0168-8278(00)80131-2
